# GM-CSF Expression and Macrophage Polarization in Joints of Undifferentiated Arthritis Patients Evolving to Rheumatoid Arthritis or Psoriatic Arthritis

**DOI:** 10.3389/fimmu.2020.613975

**Published:** 2021-02-17

**Authors:** Sara Fuentelsaz-Romero, Andrea Cuervo, Lizbeth Estrada-Capetillo, Raquel Celis, Raquel García-Campos, Julio Ramírez, Sergi Sastre, Rafael Samaniego, Amaya Puig-Kröger, Juan D. Cañete

**Affiliations:** ^1^ Unidad de Inmuno-Metabolismo e Inflamación, Instituto de Investigación Sanitaria Gregorio Marañón (IiSGM), Hospital General Universitario Gregorio Marañón, Madrid, Spain; ^2^ Unidad de Artritis, Servicio de Reumatología, Hospital Clínic and IDIBAPS, Barcelona, Spain; ^3^ Sección de Artroscopia, Servicio de Cirugía Ortopédica y Traumatología, Hospital Clínic, Barcelona, Spain; ^4^ Unidad de Microscopía Confocal, Instituto de Investigación Sanitaria Gregorio Marañón (IiSGM), Hospital General Universitario Gregorio Marañón, Madrid, Spain

**Keywords:** GM-CSF, macrophages, synovial tissue, undifferentiated arthritis, psoriatic arthritis, rheumatoid arthritis

## Abstract

**Background and Aims:**

GM-CSF-dependent macrophage polarization has been demonstrated in rheumatoid arthritis (RA). Our aim was to seek diagnostic/prognostic biomarkers for undifferentiated arthritis (UA) by analyzing GM-CSF expression and source, macrophage polarization and density in joints of patients with UA evolving to RA or PsA compared with established RA or PsA, respectively.

**Methods:**

Synovial tissue (ST) from patients with UA evolving to RA (UA>RA, n=8), PsA (UA>PsA, n=9), persistent UA (UA, n=16), established RA (n=12) and PsA (n=10), and healthy controls (n=6), were analyzed. Cell source and quantitative expression of GM-CSF and proteins associated with pro-inflammatory (GM-CSF-driven) and anti-inflammatory (M-CSF-driven) macrophage polarization (activin A, TNFα, MMP12, and CD209, respectively) were assessed in ST CD163^+^ macrophages by multicolor immunofluorescence. GM-CSF and activin A levels were also quantified in paired synovial fluid samples. CD163^+^ macrophage density was determined in all groups by immunofluorescence.

**Results:**

Synovial stromal cells (FAP^+^ CD90^+^ fibroblast, CD90^+^ endothelial cells) and CD163^+^ sublining macrophages were the sources of GM-CSF. ST CD163^+^ macrophages from all groups expressed pro-inflammatory polarization markers (activin A, TNFα, and MMP12). Expression of the M-CSF-dependent anti-inflammatory marker CD209 identified two macrophage subsets (CD163^+^ CD209^high^ and CD163^+^ CD209^low/-^). CD209^+^ macrophages were more abundant in ST from healthy controls and PsA patients, although both macrophage subtypes showed similar levels of pro-inflammatory markers in all groups. In paired synovial fluid samples, activin A was detected in all patients, with higher levels in UA>RA and RA, while GM-CSF was infrequently detected. ST CD163^+^ macrophage density was comparable between UA>RA and UA>PsA patients, but significantly higher than in persistent UA.

**Conclusions:**

GM-CSF is highly expressed by sublining CD90^+^ FAP^+^ synovial fibroblasts, CD90^+^ activated endothelium and CD163^+^ macrophages in different types of arthritis. The polarization state of ST macrophages was similar in all UA and established arthritis groups, with a predominance of pro-inflammatory GM-CSF-associated markers. CD163^+^ macrophage density was significantly higher in the UA phases of RA and PsA compared with persistent UA. Taken together, our findings support the idea that GM-CSF is a strong driver of macrophage polarization and a potential therapeutic target not only in RA but also in PsA and all types of UA.

## Introduction

Undifferentiated arthritis (UA) is frequent in early arthritis clinics, with a prevalence of 30%–50% (1–3). After 1 year of follow up, approximately 30% of patients progress to rheumatoid arthritis (RA) and 7%–15% to peripheral spondyloarthritis (pSpA), mainly to psoriatic arthritis (PsA) (4). SpA is a concept encompassing a group of musculoskeletal inflammatory diseases (axial spondyloarthritis, peripheral SpA, PsA, reactive arthritis, arthritis associated with inflammatory bowel disease) sharing immunogenetic, clinical, and radiographic characteristics. PsA is the most prevalent peripheral SpA and is characterized by skin and musculoskeletal inflammation (arthritis, enthesitis, dactylitis, and spondylitis) (5, 6).

RA and peripheral PsA are chronic inflammatory diseases mediated by the immune system and, although joint damage may be early and frequent in both diseases if not treated, their pathogenesis may be different regarding the relative implication of the adaptive and innate immune systems in each of them. This is illustrated by therapies that are useful in treating RA (anti-CD20, rituximab; CTL4-IgG, abatacept; anti- IL6 R, tocilizumab) but not PsA. Inversely, therapies inhibiting the IL-17/IL-23 pathway are successful in treating PsA, but do not work in RA patients (7).

The search for biomarkers enabling early classification of UA patients, earlier treatment and better outcomes has recently increased interest in the study of inflamed synovial tissue (ST) or synovitis, the primary target of inflammatory joint diseases (8). Synovitis in RA and PsA, the two most-frequent definite diagnoses in UA, share multiple similarities in the composition and structural distribution of their cellular infiltrates (9). CD68^+^ macrophages accumulate in the synovium of RA and PsA joints, where they show destructive and remodeling potential and contribute considerably to joint inflammation and damage (10, 11). In fact, RA and PsA macrophage density correlates with disease activity (12, 13). Sublining CD68^+^ macrophage density has been shown to be similar in PsA and RA synovitis (14) although, in a small study we found that CD68^+^ macrophage density was associated with erosive disease only in RA, suggesting that the destructive potential of ST CD68^+^ macrophages may differ between RA and PsA (15).

Studies have analyzed macrophage subsets in chronic arthritis with differing results, probably due to the markers used. Hemoglobin/haptoglobin scavenger receptor CD163-positivity was proposed as a biomarker of anti-inflammatory macrophages and was overexpressed in peripheral SpA, including PsA synovitis, whereas overexpression of pro-inflammatory macrophage markers was found in RA (16). By using surface markers (CD14, CD163, CD68, CD32, CD64, CD200R, CD80) on ST macrophages from RA and PsA patients, that study found a mixed M1-proinflammatory/M2-anti-inflammatory macrophage phenotype, with M1 predominance in RA and IL-10-expressing macrophages in SpA, including PsA (16). More recently, by using single cell technologies, Zhang et al. identified the profile of cell populations that drive joint inflammation in RA and found transcriptional heterogeneity in the synovial myeloid population (17). The function of synovial macrophages during joint homeostasis and inflammation has been also studied. Murine Trem2^+^ lining macrophages constantly repopulate from proliferating tissue resident interstitial macrophages and form a protective epithelial-like barrier that disintegrates during experimental arthritis (18). These macrophages are comparable with human TREM2^+^ lining macrophages, that exhibit anti-inflammatory and repair responses during remission in RA (19). Finally, Kuo et al. showed that sublining HBEGF^+^ inflammatory macrophages promoted fibroblast invasiveness in an epidermal growth factor receptor-dependent manner and contributes to fibroblast-mediated joint destruction (20).

The molecular characterization of GM-CSF-dependent (pro-inflammatory) and M-CSF-driven (anti-inflammatory) monocyte-derived macrophages (21–23) has allowed the definition of the polarization state of macrophages in RA. Specifically, CD163^+^ RA ST macrophages have a high expression of GM-CSF-associated markers (MMP12, EGLN3, INHBA, TNFα, and CCR2) but low levels of the M-CSF-associated marker CD209 (21–23). Given the putative pathological role of GM-CSF and M-CSF in synovitis (24–26), our aims were to seek diagnostic/prognostic biomarkers by analyzing potential differences in GM-CSF expression and its cellular source, and CD163^+^ macrophage polarization and density in joints of patients with UA evolving to RA or PsA compared with persistent UA and established RA or PsA, respectively.

## Methods

### Patients

Patients with UA and synovial biopsies were retrospectively selected when they met the classification criteria for RA or PsA during follow up. Synovial biopsies obtained by rheumatologic arthroscopy (9) from inflamed knee joints of patients with active persistent UA synovitis (UA, n=16), UA evolving to RA (UA>RA, n=8), and UA evolving to PsA (UA>PsA, n=9), together with synovial biopsies from patients fulfilling the American College of Rheumatology (ACR) criteria for RA (27) (n=12) or the CASPAR criteria for PsA (28) (n=10) as positive controls, were included. ST from six healthy controls (four men, two women, aged 36± 5 years) undergoing arthroscopic meniscectomy, were included. No patient had received biological therapy at biopsy. Arthroscopy was performed under diagnostic and/or therapeutic (lavage) indications with a 2.7 mm arthroscope (Storz, Tullingen, Germany). Eight samples were obtained from the suprapatellar pouch and the medial and lateral gutters in each patient (9). The study was approved by the Ethics Research Committee of the Hospital Clinic of Barcelona, Spain (HCB/2014/0579) and signed informed consent was obtained from each patient.

### Immunofluorescence and Multicolor Confocal Microscopy

Multicolor confocal microscopy was performed as previously described (21, 29, 30). Briefly, the following antibodies were used: FITC-labelled anti-CD163 (Ber-Mac3, MBL International Corp., MA, USA), anti-M-CSFR/CD115 (AF329, R&D Systems), anti-TNFα (ab6671, Abcam), anti-MMP12 (ab66157, Abcam), anti-INHBA (ab97705, Abcam), anti-CD209 (MR1; kindly provided by Angel L. Corbí, Centro de Investigaciones Biológicas, Madrid, Spain), anti-CD90 (E510, BD Pharmingen), anti-FAP (ab65398, Abcam), anti-GM-CSF (sc-13101, Santa Cruz Biotechnology), anti-CD3 (SK7), and anti-CD4 (SK3), from BD Biosciences, isotype-matched control antibodies, and fluorochrome-conjugated secondary antibodies (Jackson ImmunoResearch). Tissues samples were snap-frozen in OCT, and 4 μm cryosections were blocked for 10 min with 1% human immunoglobulins and incubated with primary (1-5 μg/ml) and appropriate secondary antibodies. Imaging was performed using an inverted confocal microscope (SPE, Leica Microsystems) and an ACS-APO 20x/NA 0.60 glycerol immersion objective. Similar acquisition settings were used for all samples. Considering that fluorescence intensity correlated with molecules on the surface of the cell, the mean number of molecules of every single cell (Mean Fluorescence Intensity, MFI) was calculated using ImageJ/FIJI software (National Institutes of Health, Bethesda, MD, USA). All quantifications were blinded and at least three random fields 50 µm from the lining layer were evaluated for each type of tissue, quantifying the expression of activin A, TNF-α, MMP12, and CD209 in all segmented CD163^+^ macrophages. CD115 and/or CD163 macrophage pan-markers were used to segment these cells in tissues and estimate the mean intensity of the proteins of interest. Pan-markers were intentionally acquired in saturated conditions to better depict macrophages areas. Macrophage density was normalized based on the selected tissue area (mm^2^). After background subtraction, data were plotted using GraphPad software (GraphPad Software, La Jolla, CA, USA).

### Immunohistochemistry for Pathotype Definition of Synovial Tissue

The synovial biopsies were embedded in paraffin, sectioned, and subjected to antigen retrieval by cooking when required. The slides were subsequently stained with an automated immunostainer (TechMate 500 Plus; Dako, Cambridge, UK) using the following monoclonal antibodies: anti-CD3 (clone PS1; Novocastra, Newcastle, UK), anti-CD20 (clone L26; Dako), anti-CD68 (clone KP-1; Dako), and anti-CD138 (clone B-B4; Santa Cruz Biotechnology, Inc., San Diego, CA, USA. As a negative control, the primary antibodies were substituted by isotype- and concentration-matched control antibodies. The primary antibodies were subsequently detected by an avidin-biotin-peroxidase-based method (Envision System; Dako) and an aminoethylcarbazole color reaction (Sigma-Aldrich, St. Louis, MO, USA) as described previously in detail (9). Finally, the slides were counterstained with hematoxylin. The stained slides were scored by digital image analysis by an independent observer (RC) who was blinded to diagnosis and clinical data. Each stained slide in its entirety was scored by dividing it in different regions. Within each region, the number of stained cells per area as well as the percentage of stained cells were measured in at least 20 high-power fields using the AnalySIS^®^ Imaging processing program (Olympus^®^) as described previously in detail (9).

### Macrophage Culture

Human peripheral blood mononuclear cells (PBMC) were isolated from buffy coats from normal donors over a Lymphoprep (Nycomed Pharma) gradient. Monocytes were purified from PBMC by magnetic cell sorting using CD14 microbeads (Miltenyi Biotech). Monocytes were cultured at 0.5 x 10^6^ cells/ml for 7 days in RPMI 1640 supplemented with 10% fetal calf serum, at 37°C in a humidified atmosphere with 5% CO_2_, and containing GM-CSF (1000 U/ml) to generate GM-CSF-polarized macrophages (termed GM-MØ), or M-CSF (10 ng/ml) for M-CSF-polarized macrophages (termed M-MØ). Cytokines were added every 2 days.

### Flow Cytometry

Phenotypic analysis was carried out by immunofluorescence using standard procedures. Mouse monoclonal antibodies used for cell-surface staining included FITC-labeled anti-CD14 (Miltenyi), anti-CD115 (R&D Systems) and FITC-labeled anti-CD163 (MBL International Corp., MA). Alexa Fluor-647-labeled isotype-matched secondary antibody (Jackson ImmunoResearch) was used to stain anti-CD115. Control γ_1_ FITC/γ_2a_ PE (BD) was included as a negative control.

### Quantitative Real Time RT-PCR and GSEA

Total RNA was retrotranscribed and cDNA was quantified using the Universal Human Probe Roche library (Roche Diagnostics). Quantitative real-time PCR (qRT-PCR) was performed on a LightCycler^®^ 480 (Roche Diagnostics). Assays were made in triplicate and results normalized according to the expression levels of TBP. The results were obtained using the ΔΔCT method for quantitation. The oligonucleotides used to quantify mRNA transcripts were (5´-3´): CD209s cagagtggggtgacatgagtgac; CD209as gtgaagttctgctacgcaggag; INHBAs ctcggagatcatcacgtttg; INHBAas ccttggaaatctcgaagtgc; MMP12s tgtcactaccgtgggaaataag; MMP12as aacactggtctttggtctctcag; TBPs cggctgtttaacttcgcttc; TBPas cacacgccaagaaacagtga; TNFαs cagcctcttctccttcctgat; TNFαas gccagagggctgattagaga. For Gene Set Enrichment Analysis (GSEA) (http://software.broadinstitute.org/gsea/index.jsp) (31), the gene signature from RA synovial macrophages versus monocyte-derived macrophages limma analysis of the microarray data in GSE10500 was used (31). The previously defined GM-MØ-specific markers data set (from GSE68061) was used for GSEA (32).

### ELISA

GM-CSF (Human GM-CSF ELISA MAX Deluxe, BioLegend) and activin A (Human Activin A DuoSet ELISA, R&D Systems) were quantified using commercially-available ELISA.

### Statistical Analysis

The Mann Whitney test and Student’s *t* test were used and Spearman’s correlation was determined. A value of *p<*0.05 was considered statistically significant (^*^, p<0.05; ^*^ ^*^, p<0.01; ^**^ ^*^, p<0.001). The analysis was made using GraphPad Prism software.

## Results

### Clinical and Demographic Features

Clinical, demographic and serologic data of the patients included are detailed in **Table 1**. UA>RA patients had significantly fewer swollen and tender joints, and a lower DAS28, than established RA patients. UA>PsA patients were significantly younger than those with established PsA. No significant between-group differences were found regarding systemic inflammation biomarkers. Some patients were taking csDMARDs, mostly methotrexate, at arthroscopy. A high percentage of patients did not receive csDMARDs, due to knee involvement or because they had few inflamed joints, which were treated with NSAIDs or local infiltration with glucocorticoids before 3 months before arthroscopy. No patient was treated with biological therapy before arthroscopy.

**Table 1:** Clinical, demographic and serologic data.

	UA	UA>RA	UA>PsA	RA	PsA	UA>RA vs UA>PsA	UA>RA vs RA	UA>PsA vs PsA
	n=16	n= 8	n= 9	n= 12	n=10	*p*	*p*	*p*
**Age (years)**	57 (40-65)	56 (42-71)	44 (35-49)	58 (52-71)	53 (48-62)	0.102	0.643	0.024
**Sex (male) n (%)**	5 (31)	3 (38)	5 (56)	3 (25)	8 (80)	0.457	0.550	0.252
**Disease duration until synovial biopsy (months)**	30 (21-37)	13 (6-51)	6 (3-30)	104 (73-202)	129 (45-265)	0.289	0.002	0.003
**Follow-up until definite diagnosis (months)**	32 (11-59)*	1 (0.5-7)	1 (0.5-2)	NA	NA	0.847	NA	NA
**Disease duration (months)**	60 (36-97)	34 (7-57)	6 (4-36)	28 (10-82)	25 (12-56)	0.177	0.671	0.060
**SJC**	1 (1-2)	2 (1-9)	1 (1-2)	11 (4-19)	1 (1-1)	0.141	0.033	0.850
**TJC**	1 (1-2)	3 (2-5)	1 (1-2)	19 (4-25)	1 (1-1)	0.009	0.015	0.633
**CRP basal (mg/dl)**	1.34 (0.57-2.53)	2.46 (0.51-3.12)	1.70 (0.39-8.23)	4.45 (1.20-10.10)	3.55 (0.64-7.87)	0.630	0.117	0.744
**ESR basal (mm/h)**	17 (7-32)	34 (20-67)	30 (12-40)	49 (22-69)	8 (5-41)	0.499	0.589	0.204
**DAS28 basal (VSG)**	3.34 (2.68-3.82)	4.40 (3.43-5.39)	3.73 (2.85-4.17)	5.84 (4.88-7.59)	3.13 (2.27-4.24)	0.102	0.039	0.391
**RF and/or ACPA n (%)**	0	4 (50)	0	9 (75)	0	0.006	0.710	–
**csDMARD n (%)**	7 (44)	2 (25)	6 (67)	6 (50)	2 (20)	0.205	0.667	0.040

Data are expressed as median (Interquartil range). Mann Whitney test, T student.

UA, Undifferentiated Arthritis; UA>RA, Undifferentiated Arthritis evolving to RA; UA>PsA, Undifferentiated Arthritis evolving to PsA; RA, Rheumatoid Arthritis; PsA, Psoriatic Arthritis; SJC, Swollen Joint Count; TJC, Tender Joint Count; CRP, C-Reactive Protein; ESR, Erythrocyte Sedimentation Rate; DAS, Disease Activity Score; RF, Rheumatoid Factor; ACPA, Anti-Citrullinated Protein Antibody; csDMARD, Conventional Synthetic Drugs Modifying; NA, not applicable; p, pvalue; *Follow-up in persistent UA group, without definite diagnosis.

### Synovial Tissue Stromal Cells and CD163^+^ Macrophages Are the Main Source of GM-CSF in Undifferentiated and Established Arthritis

**Figure 1:**
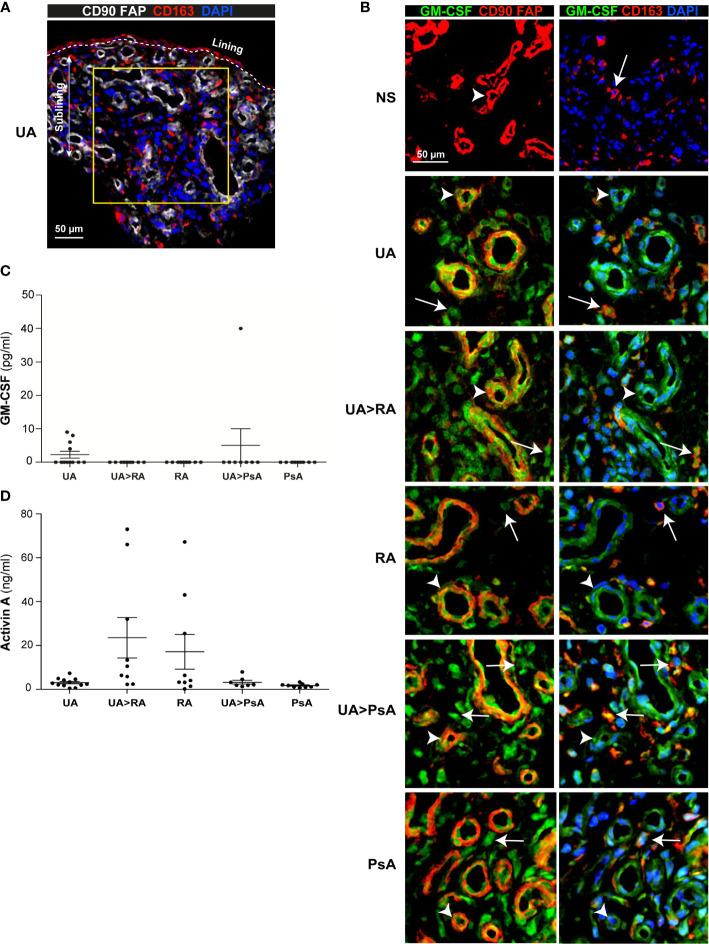
GM-CSF expression in stromal cells from synovial tissue of undifferentiated arthritis patients. **(A)** Representative immunofluorescence image of persistent undifferentiated arthritis (UA) synovial tissue (ST) determined by confocal microscopy using anti-CD90, anti-FAP (white), and anti-CD163 (red)-specific antibodies; nuclei were counterstained with DAPI. Lining and sublining layers are shown. The region of interest in the sublining layer (yellow square) is located 50 µm from the lining layer. **(B)** Immunofluorescence analysis of normal synovium (NS), persistent undifferentiated arthritis (UA), undifferentiated arthritis evolving to RA (UA>RA), rheumatoid arthritis (RA), undifferentiated arthritis evolving to psoriatic arthritis (UA>PsA) and psoriatic arthritis (PsA) ST determined by confocal microscopy using anti-GM-CSF (green), anti-CD90, anti-FAP or anti-CD163 (red)-specific antibodies; nuclei were counterstained with DAPI. Arrowheads indicate CD90^+^FAP^+^ fibroblast and white arrows indicate CD163^+^ macrophages. Samples were devoid of staining when incubated with isotype-matched irrelevant antibodies as negative controls. The experiment was carried out in independent samples from each type (NS, n=6; UA, n=16; UA>RA, n=8; RA, n=12; UA>PsA, n=9; and PsA, n=10) and representative experiments are shown. CD163^+^ macrophages are indicated by white arrows. Scale bars, 50 µm. Determination of levels of GM-CSF **(C)** and activin A **(D)** in the synovial fluid of patients with UA (n=12), UA>RA (n=9), RA (n=9), UA-PsA (n=7), and PsA (n=9), as determined by ELISA.

To assess GM-CSF levels in the undifferentiated phases of human arthritis, we first determined GM-CSF expression in the ST sublining of persistent UA, UA>RA, UA>PsA, established RA and PsA patients, and healthy controls (**Figures 1A, B**). Except in healthy ST, GM-CSF was readily detectable in all groups (**Figure 1B**). Immunofluorescence analysis revealed GM-CSF in CD90^+^ activated endothelium, CD90^+^ FAP^+^ fibroblasts from the sublining layer around the endothelium, and in sublining CD163^+^ macrophages (**Figure 1B**). However, GM-CSF was not found in CD3^+^ or CD4^+^ T cells (data not shown). These results indicate that GM-CSF is detected similarly in the earlier and established stages of RA and PsA, and in persistent UA, and that GM-CSF-producing cells are pathogenic CD90^+^ FAP^+^ synovial fibroblasts (28), CD90^+^ activated endothelium (33) and sublining CD163^+^ macrophages.

### Activin A, but Not GM-CSF, Is Increased in Synovial Fluid From All Arthritis Groups

Analysis of paired synovial fluid (SF) samples from patients with persistent UA (n=12), UA>RA (n=9), UA>PsA (n=7), RA (n=9) and PsA (n=9) indicated that GM-CSF levels were below detection levels in most SF samples, except for 4 out of 9 (44%) persistent UA patients who were csDMARD naïve, and one UA>PsA patient out of 7 analyzed (14%), who had increased GM-CSF levels (**Figure 1C**). We also measured the level of activin A, which plays an important role in the early phases of the inflammatory process (34). By contrast to GM-CSF, high levels of activin A were detected in all SF samples, with concentrations ranging from 0.2 to 72 ng/ml, with UA>RA and RA samples expressing higher levels than all other groups (**Figure 1D**). Taken together, our results suggest that ST GM-CSF and SF activin A are detected in both the undifferentiated and established phases of arthritis, but with significantly higher levels of activin A in the undifferentiated and established phases of RA.

### Synovial Tissue CD163^+^ Macrophages From Patients With Undifferentiated and Established Arthritis Exhibit a Predominantly GM-CSF-Dependent Pro-Inflammatory Polarization State

**Figure 2:**
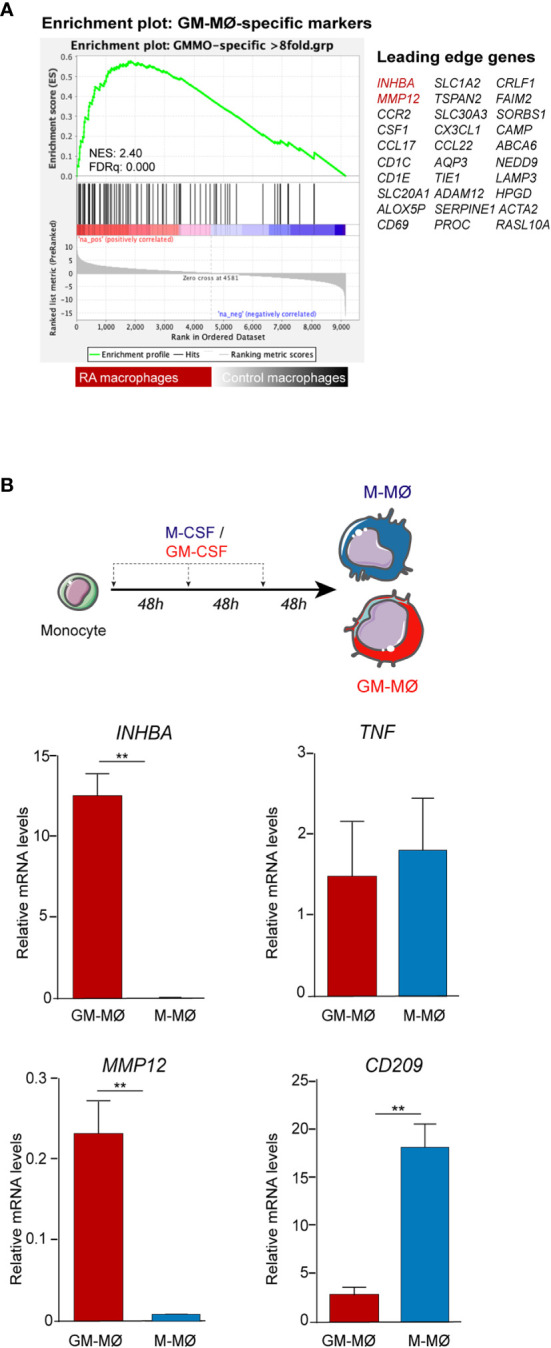
Expression of polarization markers by RA macrophages and *in-vitro* generated monocyte-derived macrophages. **(A)** Gene set enrichment analysis (GSEA) on the ranked list of genes obtained from the comparison of the transcriptome of RA macrophages (RA mac) *versus* monocyte-derived macrophages (M-MØ) (GSE10500) using the set of genes with the highest GM-CSF-induced upregulation in monocyte-derived macrophages differentiated with GM-CSF (GSE68061). **(B)** *INHBA*, *MMP12*, *CD209*, and *TNFA* mRNA expression levels determined by qRT-PCR on monocytes differentiated with GM-CSF (GM-MØ) and M-CSF (M-MØ). Mean ± SEM of four independent donors are shown (^*^ *p* < 0.05, ^**^ *p* < 0.01, Student *t* test).

**Figure 3:**
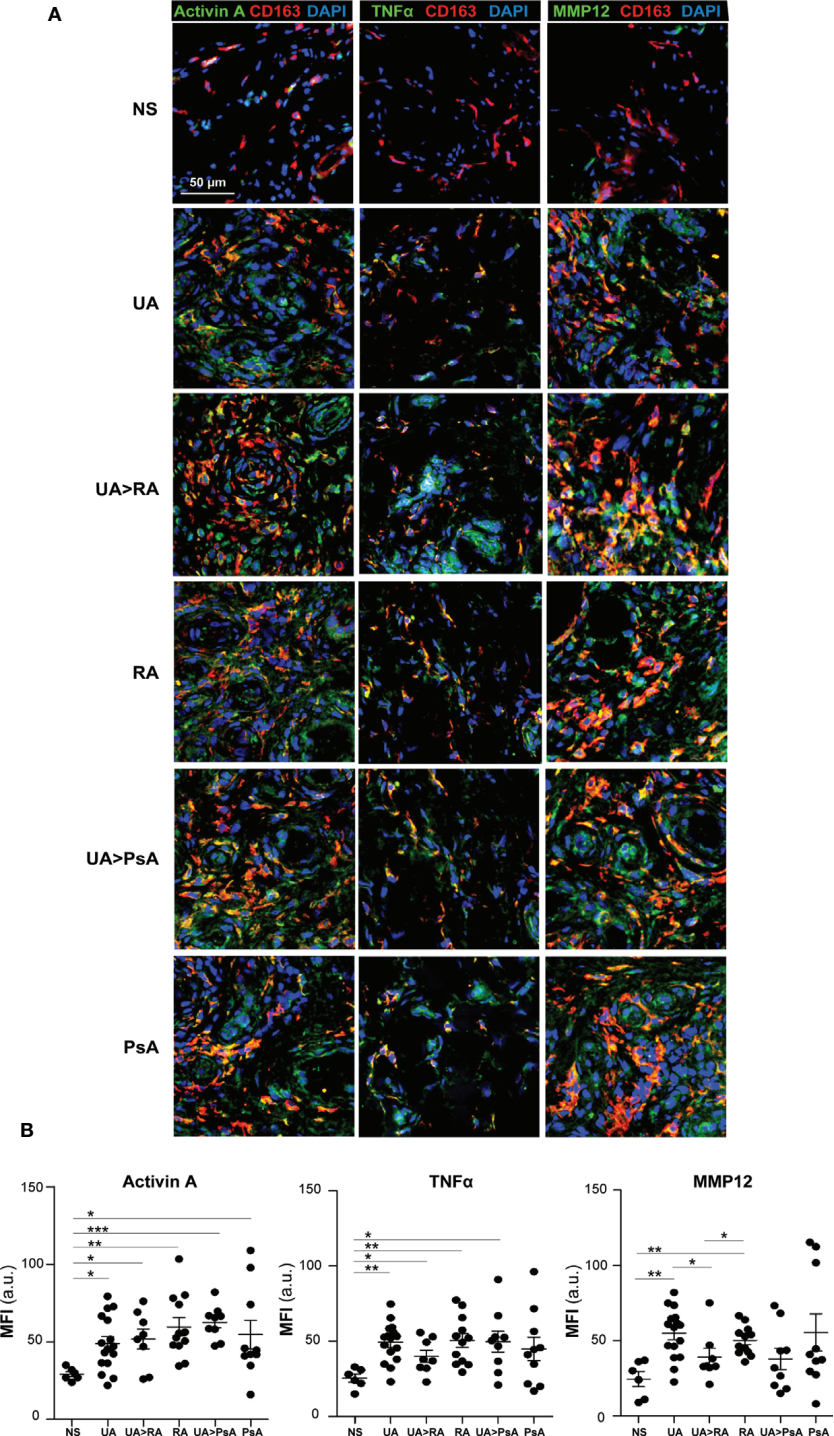
Expression of macrophage-pro-inflammatory polarization markers by CD163^+^ macrophages from undifferentiated arthritis patients. **(A)** Immunofluorescence analysis of synovial tissues as determined by confocal microscopy using anti-activin A, TNFα-, MMP12-specific antibodies; nuclei were counterstained with DAPI. Samples were devoid of staining when incubated with isotype-matched irrelevant antibodies as negative controls. The experiment was carried out in independent samples from each type (NS, n=6; UA, n=16; UA>RA, n=8; RA, n=12; UA>PsA, n=9; and PsA, n=10) and representative experiments are shown. Scale bars, 50 µm. **(B)** Summary dot plot showing mean MFI values of activin A, TNF-α and MMP12 expression in CD163^+^ macrophages from NS, UA, UA>RA, RA, UA>PsA, and PsA synovial tissues samples. Mean ± SEM are shown (^*^ *p* < 0.05, ^**^ *p* < 0.01, ***p < 0.001, Mann-Whitney test).

Gene ontology analysis using GSEA to compare RA macrophages and monocyte-derived macrophage transcriptome (31) revealed a very significant positive enrichment of pro-inflammatory GM-CSF-conditioned monocyte-derived macrophages (GM-MØ)-specific genes in RA macrophages (**Figure 2A**). In fact, the leading edge of the GSEA revealed the genes encoding activin A (*INHBA*), the metalloproteinase MMP12 and the chemokine CCL17 (35) (**Figure 2A**). Since our results indicated that ST in UA show enriched expression of GM-CSF in the stromal cells and activin A in the SF, we next determined the expression of proteins associated with GM-CSF-driven polarization (activin A, MMP12, and TNFα) and M-CSF-driven polarization (CD209) (21) (**Figure 2B**) in ST CD163^+^ macrophages of persistent UA, UA>RA, UA>PsA, and in established RA and PsA (**Figure 3A**). Similar to active established RA (21), activin A, TNFα, and MMP12 were readily found in CD163^+^ macrophages within the synovial sublining of UA, either evolving or not (**Figure 3A**), and essentially the same results were obtained in PsA, where ST CD163^+^ macrophages expressed GM-CSF-dependent polarization markers (**Figure 3A**). By contrast, CD163^+^ macrophages from healthy synovium did not express TNFα, MMP12, or activin A (**Figure 3A**). Quantification of the above indicated markers revealed that CD163^+^ macrophages from UA>RA and UA>PsA exhibited similar expression levels of activin A, TNFα and MMP12 as CD163^+^ macrophages from established RA and PsA, and higher levels than CD163^+^ macrophages from healthy synovium (**Supplementary Figure 1**, **Figure 3B**). MMP12 expression was significantly higher in persistent UA than in UA>RA, although no significant differences in markers of systemic inflammation or in the percentage of patients receiving csDMARDs were found between the two UA groups (**Table 1**). Therefore, the expression of activin A, MMP12 and TNFα designates the GM-CSF-dependent pro-inflammatory state of CD163^+^ ST macrophages in undifferentiated and established arthritis (**Figures 3A, B**).

### Two Subsets of CD163^+^ CD209 Macrophages Are Found in the Synovial Tissue From Patients With Undifferentiated and Established Arthritis

**Figure 4:**
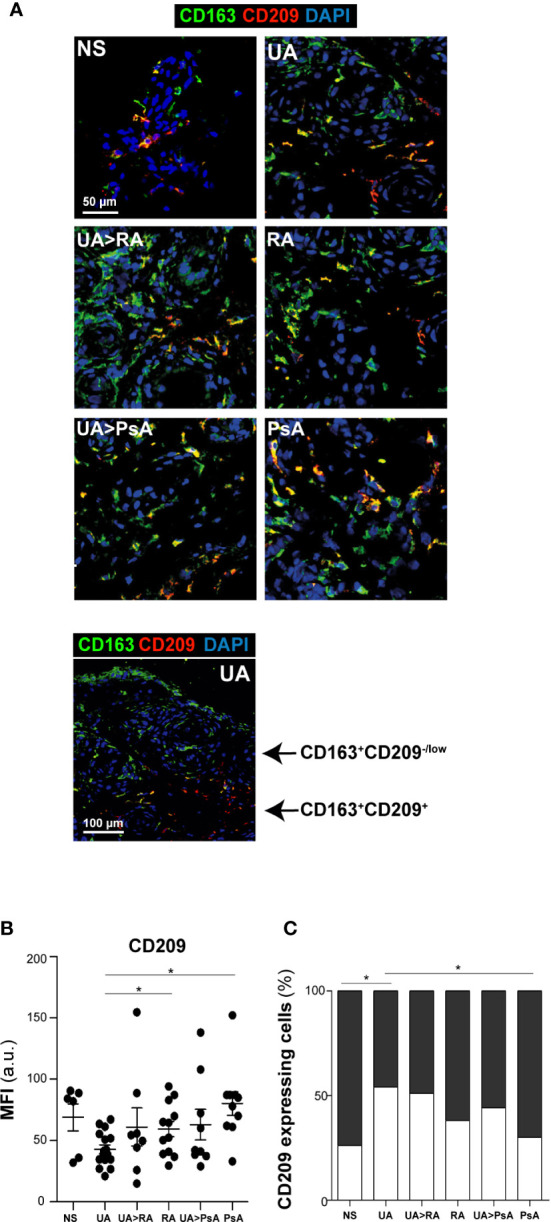
CD163^+^ CD209 macrophage subsets in synovial tissue. **(A)** CD163 (green) and CD209 (red) staining of NS, UA, UA-RA, RA, UA-PsA, and PsA synovial tissues. Scale bar, as shown. **(B)** Summary dot plot showing mean MFI values of CD209 expression in CD163^+^ macrophages from synovial tissue samples. The experiment was carried out in independent samples from each type (NS, n=6; UA, n=16; UA>RA, n=8; RA, n=12; UA>PsA, n=9; and PsA, n=10) and representative experiments are shown. Mean ± SEM are shown (^*^ *p* < 0.05, Mann-Whitney test). **(C)** Percentage of CD209-expressing macrophages in NS, UA, UA>RA, RA, UA>PsA, and PsA synovial tissues. The MFI values of eight to 12 tissues samples of each group were used for classification as “low/neg” (white) or “high” (> 34 a.u., black) CD209 expression.

With respect to the M-CSF-associated marker CD209, two subsets of CD163^+^ macrophages were found in the synovial sublining of UA and established arthritis patients (CD163^+^ CD209^+^ and CD163^+^ CD209^low/-^), which differed in their respective MFI for CD209 fluorescence (MFI >34 a.u. for the CD163^+^ CD209^+^ subset, and MFI< 34 a.u. for the CD163^+^ CD209^low/-^ subset) (**Figures 4A, B**). Synovial tissue from healthy controls also showed these two macrophage subsets, with a higher percentage of CD163^+^ CD209^+^ cells (80%) than that found in UA (**Figure 4C**). Furthermore, the percentage of the CD163^+^ CD209^+^ macrophage subset in PsA ST was significantly higher than in that from persistent UA (**Figures 4B, C**, **Supplementary Figure 2**). Taken together, these findings suggest that CD163^+^ CD209^+^ are resident macrophages with a potential anti-inflammatory function (18, 19). However, both macrophage subsets (CD209^+^ and CD209^low/-^) expressed the proinflammatory markers activin A, TNFα and MMP12 in all patients from the UA and established disease groups (**Supplementary Figure 2**).

### Macrophage Density Discriminates Undifferentiated From Established Arthritis

**Figure 5:**
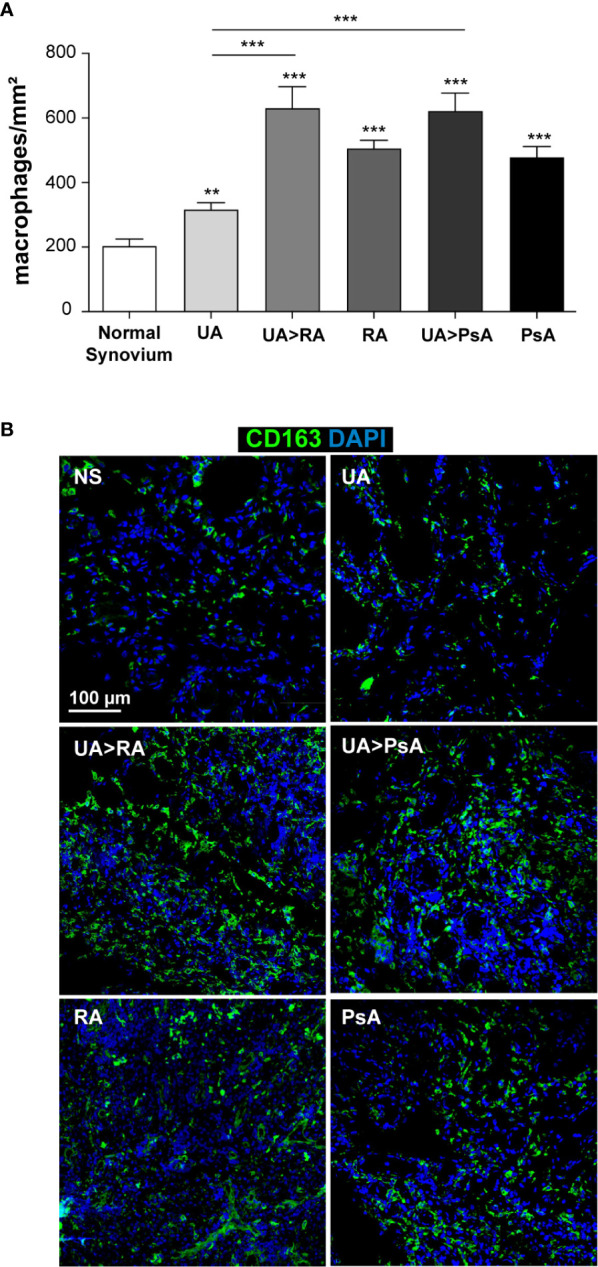
Macrophage density in normal, undifferentiated arthritis, rheumatoid arthritis and psoriatic arthritis synovial tissue. **(A)** Quantification of CD163^+^ macrophages from normal (NS, n=6), undifferentiated arthritis (UA, n=16), undifferentiated arthritis evolving to RA (UA>RA, n=8), undifferentiated arthritis becoming psoriatic arthritis (UA>PsA, n=9), rheumatoid arthritis (RA, n=12), and psoriatic arthritis (PsA, n=10) synovial tissues. The total number of macrophages was normalized based on selected tissue area (mm^2^). Mean ± SEM is shown. Significant differences are indicated (^**^ *p* < 0.01, ^***^ *p* < 0.001, Mann-Whitney test). **(B)** Immunofluorescence analysis of NS, UA, UA>RA, RA, UA>PsA, and PsA synovial tissues as determined by confocal microscopy using anti-CD163 specific antibody (green); nuclei were counterstained with DAPI (blue). The experiment was carried out in independent samples from each type (six normal synovial tissue controls and eight to 16 remaining synovial tissues) and representative experiments are shown. Scale bars, 100 µm.

We determined macrophage density in ST from patients with persistent UA, UA>RA, UA>PsA, established RA and PsA, and healthy controls. CD163 and CD115 were used to gate ST macrophages as they are expressed by most macrophages (**Supplementary Figure 3**) (36). Macrophage density was significantly higher in synovium from patients with inflammatory joint disease compared with healthy controls (203 macrophages/mm^2^) (**Figures 5A, B**) and was comparable between both UA evolving groups, with 630 macrophages/mm^2^ in UA>RA and 622 macrophages/mm^2^ in UA>PsA (**Figure 5A**). Synovial tissue from persistent UA (318 macrophages/mm^2^) showed significantly lower macrophage density than UA>RA and UA>PsA (**Figure 5A**). These results suggest that the initial phases of RA and PsA are characterized by a significantly higher density of CD163^+^ macrophages compared with persistent UA. Since all groups had a similar proportion of patients on csDMARD therapy, differences in macrophage density could not be attributed to differences in treatment. In support of this rationale, more UA>PsA patients had received csDMARDs compared with PsA patients. However, 56% of patients with persistent UA were csDMARD-naïve, and had significantly lower CD163^+^ macrophage density than UA>RA or UA>PsA (**Table 1**).

### Synovial Tissue Categorization According to Defined Pathotypes

Based on H&E and immunohistochemistry staining for CD3 (T cells), CD20 (B cells), CD138 (plasma cells), and CD68 (macrophages), synovial tissue from each UA and definite arthritis group was categorized into three distinct synovial pathotypes: lympho-myeloid, diffuse-myeloid, and pauci-immune (37). Results show that, in all the groups studied, the most frequent pathotypes were lympho-myeloid and diffuse-myeloid, ranging from 40-50% each, with the pauci-imune found in around 8% of the patients (**Supplementary Table 1**) These results are in line with those from previous studies (9, 37–40). The relative prevalence of the lympho-myeloid pathotypes in our established RA cohort, with more longstanding disease, was higher than in its undifferentiated phase (75% vs. 50%) (**Supplementary Table 1**), but the low number of cases preclude to extract further conclusion.

## Discussion

Synovial tissue macrophages are key producers of cytokines relevant to the physiopathology of chronic arthritis and their changes correlate with disease activity, radiographic progression, and response to therapy (10–13). However, macrophages are composed of a heterogeneous population with different functional profiles: pro-inflammatory, anti-inflammatory or reparative (17–20). Macrophage polarization has been demonstrated in experimental models of immune-mediated inflammatory diseases and in human cancer (41).

The characterization of ex-vivo CD14^+^ macrophages isolated from synovial fluid of patients with active RA indicate that they exhibit a transcriptomic and protein profile compatible with GM-CSF-skewed macrophage polarization (21). Activin A and other proteins encoded by several GM-CSF-associated gene markers, including MMP12 and CCR2, have been also detected in macrophages from active RA joints (21–23). GM-CSF induces macrophage differentiation and survival from hematopoietic progenitor cells (35, 42). In contrast to M-CSF, GM-CSF is undetectable in the systemic circulation during homeostasis but is produced and active at sites of tissue inflammation, and is a key driver of tissue inflammation and arthritic pain (43, 44). GM-CSF was also pathogenic in experimental models of SpA (45) and is emerging as a central player in chronic inflammatory diseases, including mouse models of multiple sclerosis (46), bowel inflammation (47), and RA (48). GM-CSF signals through GM-CSFR, priming myeloid cells to produce inflammatory mediators that promote the activation of synovial fibroblasts, activation of the vasculature and differentiation of effector T cells. Activated synovial fibroblasts secrete GM-CSF in response to inflammatory mediators, thus implying that GM-CSF is a communication conduit between the innate and adaptive immunity critically-involved in chronic inflammation (43).

The GM-CSF receptor was reported to be overexpressed in CD68^+^ and CD163^+^ macrophages from the ST of RA and PsA patients compared with osteoarthritis patients and healthy controls (49). Clinical trials targeting GM-CSF or GM-CSF receptor-α in RA have shown sustained clinical responses without major safety concerns (50, 51).

To our knowledge, this is the first study assessing GM-CSF expression and cellular source, macrophage polarization state and density in the ST of the most prevalent immune-mediated inflammatory joint diseases, RA and PsA, and their undifferentiated phases. We found that the main cellular sources of GM-CSF in the synovium of all chronic arthritis groups studied are stromal cells and CD163^+^ macrophages. Sublining CD90^+^ FAP^+^ synovial fibroblasts, the only subtype reported as pathogenic, which is specifically increased in RA compared with osteoarthritis (33) and CD90^+^ activated endothelium (52) show high GM-CSF expression. Although intracellular GM-CSF expression by ST polyfunctional lymphocytes (Th1, Th17, and ex-Th17 subtypes) have been reported using flow cytometry in PsA and the number of these cells correlated with the disease activity of psoriatic arthritis score (DAPSA) (53), we could not confirm GM-CSF expression by CD3^+^ or CD4^+^ T cells in our samples. No expression of GM-CSF on healthy synovium was found.

Among the GM-CSF-derived macrophage markers, we detected activin A both in the synovial stroma and synovial fluid of all UA and established arthritis patients, although higher levels of SF activin A were found in RA and its undifferentiated phase. Activin A is a member of the TGFβ factor family encoded by the *INHBA* gene and mediates, in part, the macrophage-polarizing ability of RA synovial fluid (21, 54). Activin A contributes to the proinflammatory macrophage polarization triggered by GM-CSF and limits acquisition of the anti-inflammatory phenotype (22). Activin A is elevated in the early stages of other inflammatory diseases and exerts pro- or anti-inflammatory activities depending on its concentration and cellular context (55). In the particular case of the undifferentiated and established arthritis in our study, activin A levels fell within the pro-inflammatory side (55), stressing its contribution to the polarization of macrophages within the inflamed synovium.

Despite the high and generalized expression of GM-CSF in the synovium of all arthritis groups analyzed, only a few csDMARD naïve patients with persistent UA had detectable levels in synovial fluid. This finding was expected, as it is known that GM-CSF is mainly expressed in inflamed tissue.

Our results confirm our previous findings in RA (21) and extend them to PsA synovitis and their respective UA phases, including persistent UA, suggesting that the pattern of polarization of ST macrophages, mainly proinflammatory, is similar and is already present in all phases of these chronic inflammatory joint diseases. A previous study analyzing macrophage subsets in human arthritis found M1 polarization predominating in RA and IL-10-expressing macrophages in SpA, including PsA (56).

Despite the pathogenic differences between RA and PsA, we found similar patterns of expression of proteins induced by GM-CSF and M-CSF in CD163^+^ macrophages of PsA, RA, and their undifferentiated phases. Although PsA ST had significantly more CD163^+^ macrophages expressing the anti-inflammatory CD209 marker, which suggests CD163^+^ CD209^+^ could represent reparative and anti-inflammatory tissue-resident macrophages (18), no differences were found in the expression of proinflammatory markers (activin A, TNF, MMP-12) between CD163^+^ macrophages CD209^+^ and CD209^low/-^.

Our results show that the density of CD163^+^ macrophages is significantly higher in UA evolving to RA or PsA compared with persistent UA. This is an unexpected finding as more UA>PsA patients had received csDMARDs therapy than PsA patients. However, 56% of patients with persistent UA were csDMARD-naïve and had significantly lower CD163^+^ macrophage density than UA>RA, UA>PsA, or established RA or PsA patients. Although cautiously, these findings suggest that a significant increase in ST CD163^+^ macrophage density could be a marker of transition from undifferentiated to definite disease, both in RA and PsA. In fact, a study searching for ST predictors of clinical differentiation in patients with seronegative undifferentiated peripheral arthritis reported that 6 out of 42 patients with a definite diagnosis after 1 year of follow up (2 RA, 2 SpA, 2 PsA) had higher histological CD68^+^ macrophage scores than patients persisting with undifferentiated arthritis (57).

Several articles using single cell technologies (17–20) have recently reported new biomarkers to clearly discriminate pro-inflammatory from anti-inflammatory, reparative macrophages. Specifically, Alivernini et al. showed that not all synovial tissue macrophages (STM) are inflammatory (MerTK^neg^) and that sustained disease remission in RA is actively maintained by MerTK^pos^ macrophages, which are also predominant in healthy synovium. If validated with additional cohorts of patients with RA in remission, the ratio of MerTK^pos^ to MerTK^neg^ STMs could be useful as a predictor of disease flare versus sustained remission to help with treatment decisions in clinical practice (19).

Our study has some limitations, including the retrospective design and the relatively small sample size. Although some patients were receiving csDMARDs (mainly methotrexate), which could modify our findings, this factor was comparable in all groups. The study has the strength of the follow-up of UA patients until they met RA or PsA classification criteria, the group of persistent UA patients (treated or not with csDMARDs) as positive controls and the ST from healthy controls. Likewise, the results were consistent for all types of arthritis.

## Conclusion

Our results show a high, generalized expression of GM-CSF in stromal cells and sublining CD163^+^ macrophages from synovial tissue of UA patients evolving or not to RA or PsA compared with healthy controls. CD163^+^ macrophages expressed a pro-inflammatory profile of biomarkers (activin A, TNFα, and MMP-12) in synovial tissue from all arthritis subtypes studied, while in paired samples of synovial fluid only activin A levels were widely detected. CD163^+^ macrophages were significantly higher in the UA phases of RA and PsA compared with persistent UA. Taken together, our findings support the concept that GM-CSF is a strong driver of macrophage polarization and a potential therapeutic target, not only in RA but also in PsA and in UA. We also found that the ST sublining of undifferentiated and established arthritis patients showed a high percentage of CD163^+^ CD209^low/-^ whereas healthy controls exhibited a high percentage of CD163^+^ CD209^+^, suggesting they may be resident macrophages with a potential anti-inflammatory function. Further studies using single cell technologies will definitively characterize phenotypes and function of synovial macrophages in undifferentiated arthritis.

## Data Availability Statement

The raw data supporting the conclusions of this article will be made available by the authors, without undue reservation.

## Ethics Statement

The study was approved by the Ethics Research Committee of the Hospital Clinic of Barcelona, Spain (HCB/2014/0579) and signed informed consent was obtained from each patient. The patients/participants provided their written informed consent to participate in this study.

## Author Contributions

AP-K and JC had full access to all the data in the study and take responsibility for the integrity of the data and the accuracy of data analysis. AP-K and JC were responsible for the study design. AC, RC, and JR performed clinical data acquisition. SF-R, LE-C, RS, RG-C, and AP-K were responsible for the analysis. SF-R, LE-C, AC, RC, AP-K, and JC performed the data interpretation. Manuscript preparation was by SF-R, LE-C, AC, AP-K, and JC. All authors contributed to the article and approved the submitted version.

## Funding

This work was supported by grants PI17/00037 from Instituto de Salud Carlos III/FEDER to AP-K, grants PI14/00785 and PI17/00993 from Instituto de Salud Carlos III to JC, grant RIER RD16/0012/0010 and RD16/0012/0007 FEDER to JC and AP-K, respectively, and co-financed by the European Regional Development Fund “A way to achieve Europe” (ERDF). SF-R is supported by a contract from Instituto de Salud Carlos III (FI18/00109) and AP-K is supported by FIBHGM. AC received grants from the Hospital Clinic of Barcelona “Emili Letang 2014”, and the Catalan Society of Rheumatology.

## Conflict of Interest

The authors declare that the research was conducted in the absence of any commercial or financial relationships that could be construed as a potential conflict of interest.

## Abbreviations

ACPA, Anti-Citrullinated Protein Antibodies; a.u., Arbitrary units; csDMARD, Conventional Synthetic Disease-Modifying Antirheumatic Drug; CRP, C-Reactive Protein; DAPI, 4′,6-diamidino-2-phenylindole; DMARDs, Disease-Modifying Antirheumatic Drugs; ESR, Erythrocyte Sedimentation Rate; FAP, Fibroblast Activation Protein; GM-CSF, Granulocyte-Macrophage Colony-Stimulating Factor; GM-CSFR, Granulocyte-Macrophage Colony Stimulating Factor Receptor Low-Affinity Subunit; INHBA, Inhibin Subunit Beta A; MFI, Mean Fluorescence Intensity; MMP12, Matrix Metalloproteinase 12; PsA, Psoriatic Arthritis; RA, Rheumatoid Arthritis; RF, Rheumatoid Factor; SEM, Standard Error of Mean; SF, Synovial Fluid; SJC, Swollen Joint Count; SL, Sublining; SpA, Spondyloarthropathy; ST, Synovial Tissue; TJC, Tender Joint Count; TNFα, Tumor Necrosis Factor Alpha; UA, Undifferentiated Arthritis; UA>RA, UA evolving to RA after follow-up; UA>PsA, UA evolving to PsA after follow-up.
