# Supplementary figures and images for: GM-CSF Expression and Macrophage Polarization in Joints of Undifferentiated Arthritis Patients Evolving to Rheumatoid Arthritis or Psoriatic Arthritis
Source: Front Immunol. 2021 Feb 17;11:613975. doi: 10.3389/fimmu.2020.613975 (PMC7925849; doi:10.3389/fimmu.2020.613975)

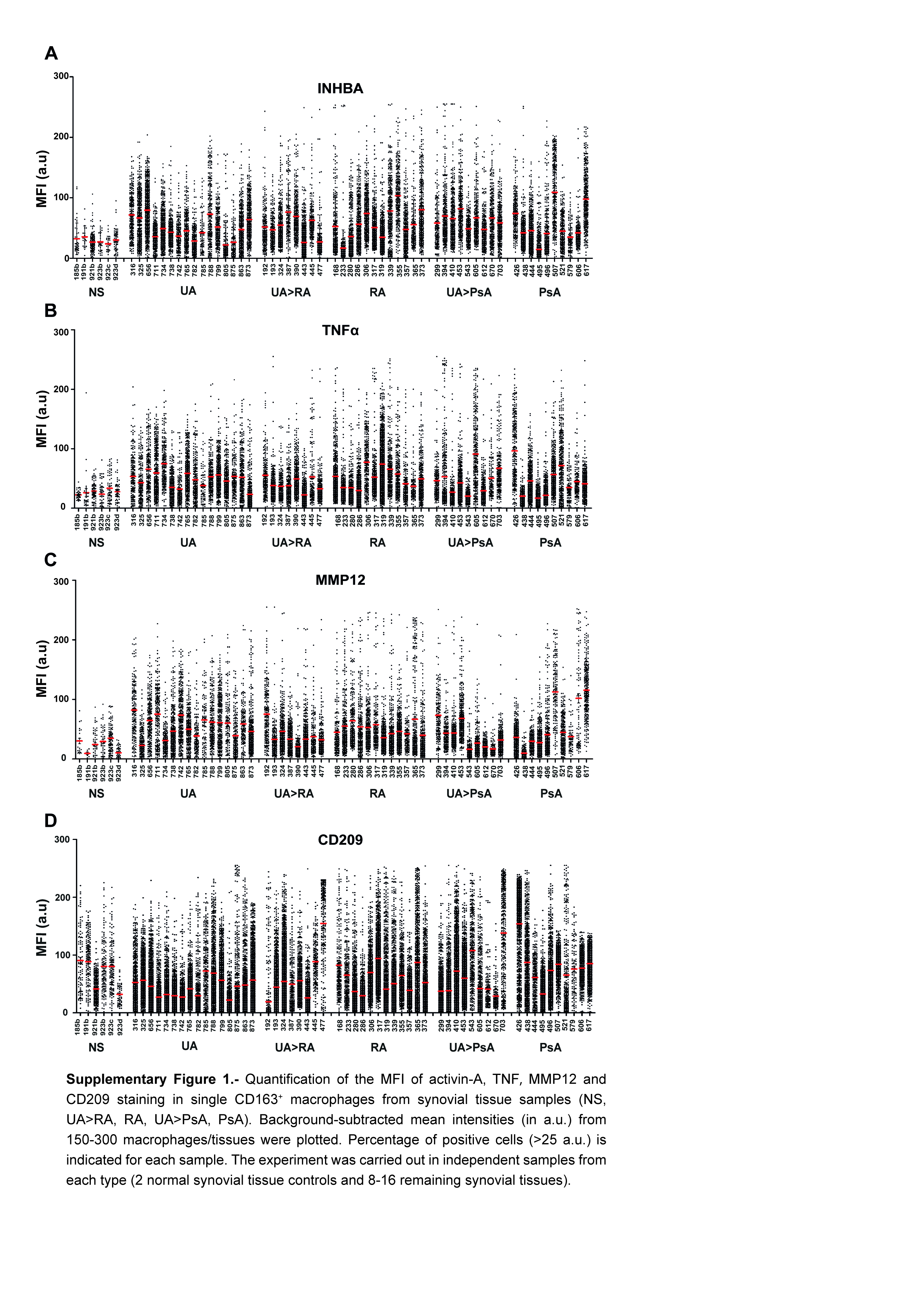

Supplement: Supplementary file 1 [file Image_1.tif]

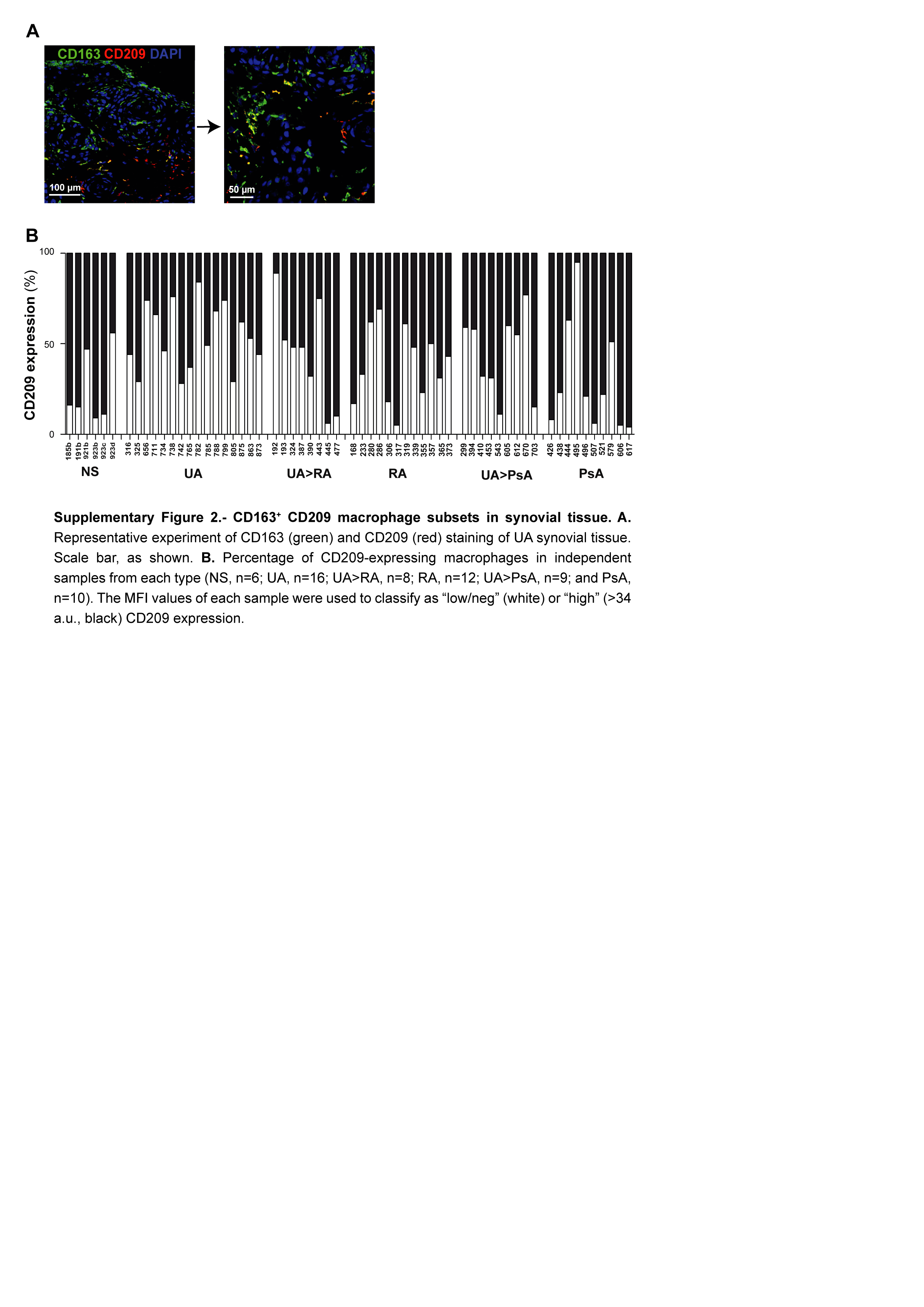

Supplement: Supplementary file 2 [file Image_2.tif]

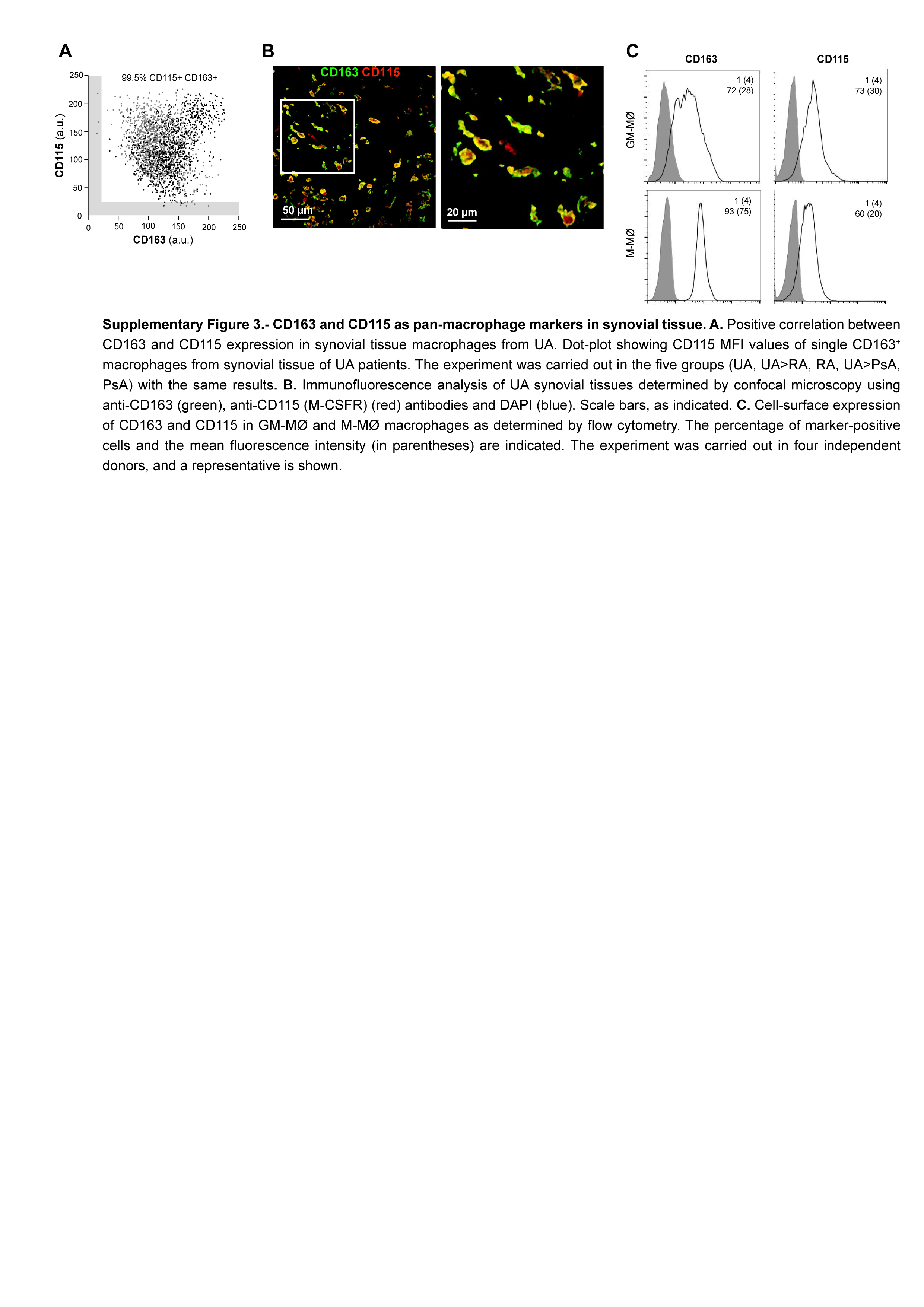

Supplement: Supplementary file 3 [file Image_3.tif]
