# Supplementary material for: GM-CSF Expression and Macrophage Polarization in Joints of Undifferentiated Arthritis Patients Evolving to Rheumatoid Arthritis or Psoriatic Arthritis
Source: Front Immunol. 2021 Feb 17;11:613975. doi: 10.3389/fimmu.2020.613975 (PMC7925849; doi:10.3389/fimmu.2020.613975)
Supplement: Supplementary file 4 [file Table_1.pdf]

Pathotypes: n (%)	UA naïve<br><br>n=7	UA + csDMARDs<br><br>n=9	UA>RA<br><br>n=8	UA>PsA<br><br>n=9	RA<br><br>n=12	PsA<br><br>n= 10
**Lympho-Myeloid**	3 (42.9)	3 (33.4)	4 (50)	4 (44.5)	9 (75)	4 (40)
**Difusse-Myeloid**	4 (57.1)	5 (55.6)	3 (37.5)	4 (44.5)	2 (16.6)	5 (50)
**Pauci-immune**	0	1 (11)	1 (12.5)	1 (11)	1 (8.4)	1 (10)

Supplementary Table 1. Synovial pathotypes.

UA: Undifferentiated Arthritis (naïve and treated con conventional synthetic DMARDs); UA>RA: Undifferentiated Arthritis evolving to RA after the follow-up; UA>PsA: Undifferentiated Arthritis evolving to PsA after the follow-up. RA: Rheumatoid Arthritis; PsA: Psoriatic Arthritis.
